# Sex-specific ventricular morphology, function, and tissue characteristics in arterial hypertension: a magnetic resonance study of the Hamburg city health cohort

**DOI:** 10.1007/s00330-024-10797-2

**Published:** 2024-05-31

**Authors:** Jennifer Erley, Charlotte M. Jahnke, Samuel Schüttler, Isabel Molwitz, Hang Chen, Mathias Meyer, Kai Muellerleile, Ersin Cavus, Gunnar K. Lund, Stefan Blankenberg, Gerhard Adam, Enver Tahir

**Affiliations:** 1 Department of Diagnostic and Interventional Radiology and Nuclear Medicine, University Medical Center Hamburg-Eppendorf, Hamburg, Germany; 2 Department of Cardiology, University Heart and Vascular Center Hamburg Eppendorf, Hamburg, Germany; 3 Deutsches Zentrum für Herz-Kreislauf-Forschung e.V. (DZHK, German Center for Cardiovascular Research), Partner Site Hamburg/Kiel/Lübeck, Germany, Hamburg, Germany

**Keywords:** Hypertension, Cardiac magnetic resonance imaging, Sex, Risk factors

## Abstract

**Objective:**

To determine the influence of arterial hypertension (AHT), sex, and the interaction between both left- and right ventricular (LV, RV) morphology, function, and tissue characteristics.

**Methods:**

The Hamburg City Health Study (HCHS) is a population-based, prospective, monocentric study. 1972 individuals without a history of cardiac diseases/ interventions underwent 3 T cardiac MR imaging (CMR). Generalized linear models were conducted, including AHT, sex (and the interaction if significant), age, body mass index, place of birth, diabetes mellitus, smoking, hyperlipoproteinemia, atrial fibrillation, and medication.

**Results:**

Of 1972 subjects, 68% suffered from AHT. 42% with AHT and 49% controls were female. Females overall showed a higher ejection fraction (EF) (LV: regression coefficient +2.4% [95% confidence interval: 1.7; 3.1]), lower volumes and LV mass (−19.8% [−21.3; −18.5]), and prolonged native septal T1 (+22.1 ms [18.3; 25.9])/T2 relaxation times (+1.1 ms [0.9; 1.3]) (all *p* < 0.001) compared to males. Subjects with AHT showed a higher EF (LV: +1.2% [0.3; 2.0], *p* = 0.009) and LV mass (+6.6% [4.3; 9.0], *p* < 0.001) than controls. The interaction between sex and AHT influenced mapping. After excluding segments with LGE, males (−0.7 ms [−1.0; −0.3 | ) and females with AHT (−1.1 ms [−1.6; −0.6]) showed shorter T2 relaxation times than the sex-respective controls (*p* < 0.001), but the effect was stronger in females.

**Conclusion:**

In the HCHS, female and male subjects with AHT likewise showed a higher EF and LV mass than controls, independent of sex. However, differences in tissue characteristics between subjects with AHT and controls appeared to be sex-specific.

**Clinical relevance statement:**

The interaction between sex and cardiac risk factors is an underestimated factor that should be considered when comparing tissue characteristics between hypertensive subjects and controls, and when establishing cut-off values for normal and pathological relaxation times.

**Key Points:**

- *There are sex-dependent differences in arterial hypertension, but it is unclear if cardiac MR parameters are sex-specific*.
- *Differences in cardiac MR parameters between hypertensive subjects and healthy controls appeared to be sex-specific for tissue characteristics*.
- *Sex needs to be considered when comparing tissue characteristics in patients with arterial hypertension to healthy controls*.

**Graphical Abstract:**

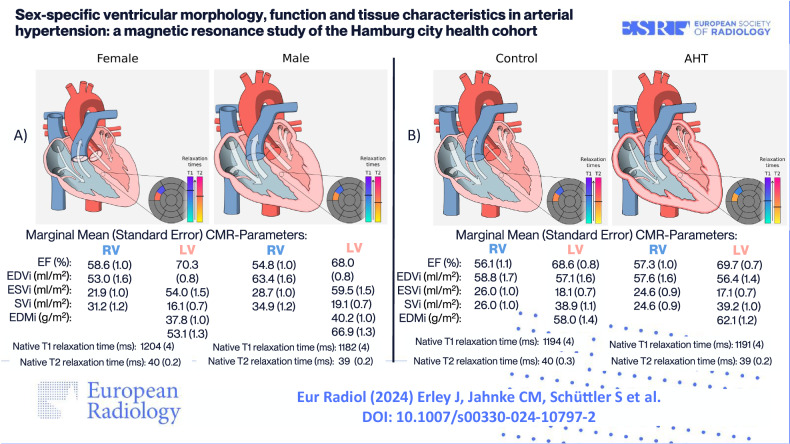

## Introduction

Arterial hypertension (AHT) is the most common modifiable risk factor for the development of heart failure [1]. The epidemiology, pathophysiology, and treatment response to AHT are sex-dependent [2]. Females have lower blood pressure values than males during early adulthood, but a proportionally greater increase in blood pressure hereafter, resulting in higher blood pressure values than males from the seventh decade onward [3, 4]. Accordingly, the prevalence of AHT is low in pre-menopausal females, but higher than in males from menopause onwards [5]. In addition, despite taking more medication than males, females are less likely to achieve blood pressure control [6]. Also, it is known that females are at a higher risk of developing heart failure with preserved EF than males in the setting of risk factors, such as AHT [7]. Nevertheless, it is uncertain if AHT-related changes in cardiac structure, function and tissue characteristics are sex-specific. Cardiac magnetic resonance imaging (CMR) is the gold standard for quantification of myocardial function and volumes and for non-invasive tissue analysis [8]. The aim of this study was to analyze the effect of sex, AHT, and the interaction between these variables on CMR measurements of cardiac structure and function, multiparametric mapping and late gadolinium enhancement (LGE).

## Materials and methods

### The Hamburg City Health Study (HCHS)

The Hamburg City Health Study (HCHS) is a prospective, population-based, single-center cohort-study, which was approved by the local ethics committee (Ärztekammer Hamburg, PV5131). Written informed consent was obtained from all study participants and all analyses were conducted in accordance with the Declaration of Helsinki and in compliance with local ethical guidelines. The study design has been recently published by Jagodzinski et al [9]. The first 10,000 subjects were enrolled between 2016 and 2019 and CMR was performed in 2589 subjects.

### Study population

In this subgroup analysis, female, and male individuals with AHT and non-hypertensive controls, who received a CMR exam, were investigated. AHT was defined as an average blood pressure of $\ge$ 140/90 mmHg after three blood pressure measurements were taken seated or lying down [9, 10], or as the use of $\ge$ 1 antihypertensive medication. Participants with a history of cardiac diseases, such as valvular diseases (known or diagnosed on CMR), coronary artery disease, previous myocardial infarction, previous cardiac intervention, previous cardiac surgery, and heart failure were excluded from the analysis.

### CMR imaging

CMR was performed on a 3-T scanner (MAGNETOM Skyra, Siemens Healthineers). Details of the CMR protocol have been previously published by Bohnen et al [11]. The CMR protocol included a balanced steady-state free-precession cine-CMR sequence in 2-, 3-, 4-chamber, and short-axis views, a modified Look-Locker inversion recovery sequence for native T1 mapping (three short-axis views), and a T2 prepared fast-low-angle shot sequence (FLASH) for native T2 mapping (three short-axis views) [11]. If study participants consented to the use of a contrast agent, a dose of 0.15 mmol/kg gadoterate meglumine (Dotarem, Guerbet) was administered intravenously. LGE images were acquired using a phase-sensitive inversion recovery sequence at 10–15 min after administration of the contrast agent (2-, 3-, 4-chamber and short-axis views) [11].

### CMR post-processing

The post-processing and data analysis workflow was previously described in detail [11]. The CMR-analyses of the HCH study are performed by radiologists or cardiologists with at least 2 years of experience under supervision by a SCMR/European Association of Cardiovascular Imaging (EACVI) level III approved radiologist or cardiologist. Every fifth study is re-analyzed by a second, blinded observer. For this specific analysis, data on left ventricular (LV) and right ventricular (RV) EF, cardiac output, mass, and volumes were obtained. The measurements of cardiac output, mass, and volumes were indexed to the body surface area, resulting in the following variables: end-diastolic mass index (EDMi), cardiac index (CI), end-diastolic volume index (EDVi), end-systolic volume index (ESVi) and stroke volume index (SVi). Data on midventricular native septal myocardial T1 and T2 relaxation times were acquired from the corresponding short-axis slice [11]. Binary outcomes (yes/no-answers) were gathered on the presence of LGE. Post-processing was performed using Cvi42 (Circle Cardiovascular Imaging Inc.). Data on T1 and T2 mapping in the HCH-cohort has been previously published by Cavus et al [12].

### Statistical analysis

The acquired data was tested for normality using histograms and the Shapiro-Wilk-Test. Continuous demographic data is represented using mean ($\pm$ standard deviation) or median (interquartile range), as appropriate. Demographic data was compared using student’s *t*-test for independent samples or Mann-Whitney-*U*-Test, depending on normality. Categorical demographic data was compared using chi-square test and is represented in numbers (percentage). Regarding normally distributed dependent data, generalized linear models (with link function if appropriate) were conducted including sex, AHT, the interaction between sex and AHT (if statistically significant), and a list of other variables as potential confounders: age, body mass index (BMI), place of birth (classified into European/non-European countries as dummy variables), diabetes mellitus, smoking, hyperlipoproteinemia, atrial fibrillation, and medication (classified into lipid-lowering/antihypertensive/antidiabetic/anticoagulant). If outcome data was not normally distributed, log transformation was performed to achieve normality, and log-linear models were performed. Binominal outcome variables were assessed using binary logistic generalized linear models. All complete cases were analyzed for each variable and the number of complete cases was indicated for each analysis. The models were adjusted for multiple testing using the Bonferroni correction. A *p*-value of $\le$ 0.05 was considered significant in two-tailored tests. Statistical analyses were conducted using SPSS (Version 28.0.1.1, IBM).

## Results

**Fig. 1:**
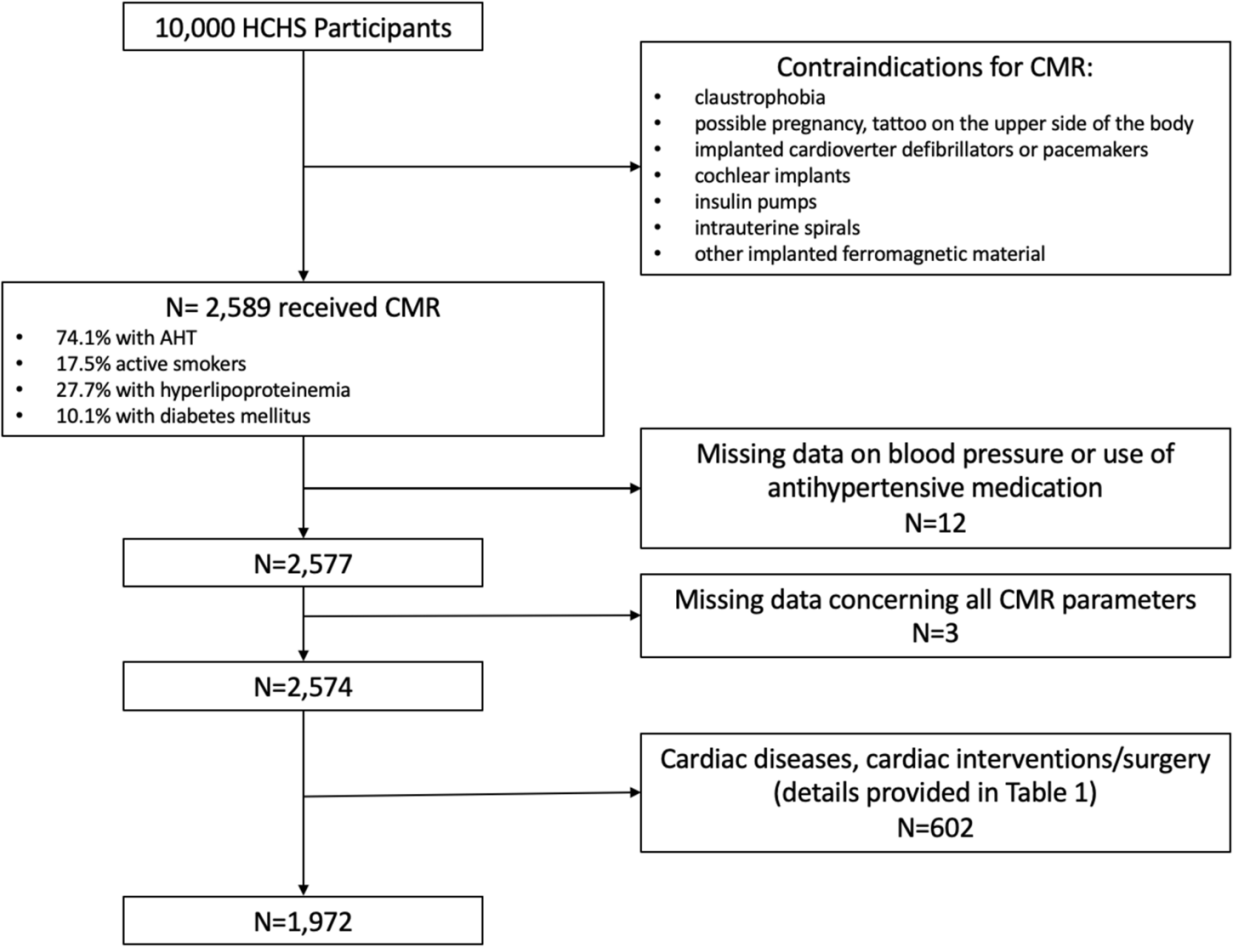
Flowchart of the in- and exclusion criteria used to derive at the final study population

Of the 2589 subjects who received a CMR exam, data on blood pressure or use of antihypertensive medication was available in 2577 subjects. 602 participants were excluded due to known cardiac diseases, known cardiac interventions/surgery, or valvular diseases (known or detected during CMR), leaving 1972 subjects for the final analysis. Of these, 875 (44.4%) received intravenous contrast agents (42.8% of controls and 45.1% of hypertensive subjects). Table 1 gives an overview of the exclusion criteria and Fig. 1 provides a flowchart of the inclusion process.

**Table 1:** Overview of the exclusion criteria and number of study participants (*n*, %) of the 602 participants excluded

Exclusion criterion	*N*	%
Valvular disease (on CMR or previously known)	217	36.0
Coronary artery disease	150	24.9
Percutaneous coronary angiography or balloon dilatation	106	17.6
Myocardial infarction	97	16.1
Heart failure	80	13.3
Coronary artery bypass surgery	18	3.0
Heart valve surgery	11	1.8
Endocarditis	11	1.8
Myocarditis	28	4.7
Atrial fibrillation	122	20.3
Percutaneous coronary angiography or balloon dilatation + coronary artery bypass surgery	4	0.7
Pacemaker or defibrillator-implantation	4	0.7
Percutaneous coronary angiography or balloon dilatation + defibrillator-implantation	1	0.2

Multiple mentions were possible

Table 2 shows the demographic characteristics of the study population, Table 3 shows the demographic features of the study population (with and without AHT), separated by sex.

**Table 2:** Comparison of demographic characteristics and CMR parameters between study participants with AHT and the control group without AHT

	Subjets with AHT (*n* = 1341)	Control group (*n* = 631)	*p*
Clinical Parameters			
Age (years)	67 (60–72) *N* = 631	60 (53–68) *N* = 1341	< 0.001
BMI (kg/m^2^)	27.0 (24.5–30.0) *N* = 1283	24.8 (22.6–27.5) *N* = 603	< 0.001
Resting heart rate (bpm)	69 (62–77) *N* = 670	67 (60–74) *N* = 300	< 0.001
Systolic resting blood pressure (mmHg) (*n*)	149 (18) *N* = 710	126 (10) *N* = 309	< 0.001
Diastolic resting blood pressure (mmHg) (*n*)	85 (10) *N* = 703	77 (7) *N* = 313	< 0.001
Female Sex	569 (42.4) *N* = 1341	307 (48.7) *N* = 631	0.009
Place of birth (European country)	1228 (91.6) *N* = 1341	571 (90.5) *N* = 631	0.428
Cardiovascular Risk Factors			
Diabetes mellitus	144 (11.4) *N* = 144	16 (2.8) *N* = 573	< 0.001
Active Smoking	217 (16.2) *N* = 1336	*N* = 135 (21.4) *N* = 631	0.005
Hyperlipoproteinemia	318 (25.1) *N* = 1267	82 (14.2) *N* = 578	< 0.001
Medication			
Intake of Medication	1093 (83.8) *N* = 1305	413 (69.9) *N* = 591	< 0.001
Lipid-lowering Medication, *n*	240 (18.4) *N* = 1305	43 (7.3) *N* = 591	< 0.001
Antihypertensive Medication, *n*	587 (45.0) *N* = 1305	0 *N* = 631	< 0.001
Antidiabetic Medication, *n*	104 (8.0) *N* = 1305	11 (1.9) *N* = 591	< 0.001
Anticoagulant Medication, *n*	172 (13.2) *N* = 1305	30 (5.1) *N* = 591	< 0.001
Laboratory Parameters			
Hemoglobin (g/dL) (*n*)	14.5 (1.1) *N* = 1302	14.3 (1.1) *N* = 618	< 0.001
Hematocrit (vol%) (*n*)	43.5 (12.1) *N* = 1302	42.7 (3.3) *N* = 618	0.023
GFR (mL/min)	87.7 (80.1–93.3) *N* = 1238	91.3 (84.6–97.2) *N* = 581	< 0.001
Glucose (mg/dL)	95.0 (88.0–103.0) *N* = 1301	90.0 (85.0–96.0) *N* = 616	< 0.001
HbA1c (%)	5.6 (5.4–5.9) *N* = 1297	5.5 (5.3–5.7) *N* = 617	< 0.001
Cholesterol (mg/dL) (*n*)	211.5 (41.8) *N* = 1307	213.1 (38.6) *N* = 616	0.129
HDL (mg/dL)	60.0 (50.0–74.0) *N* = 1307	66.0 (53.0–80.0) *N* = 616	< 0.001
LDL (mg/dL) (*n*)	123.9 (36.9) *N* = 1292	124.6 (34.5) *N* = 612	0.525
Triglycerides (mg/dL)	106.5 (80.0–146.0) *N* = 1306	90.0 (67.0–126.0) *N* = 615	< 0.001
Nt-proBNP (ng/L)	83.0 (47.0–151.3) *N* = 1310	67.0 (39.0–109.0) *N* = 614	< 0.001
Troponin (pg/mL)	2.6 (1.8–4.0) *N* = 1243	1.9 (1.2–2.8) *N* = 583	< 0.001

Numbers are mean (± SD) or median (interquartile range) for continuous and *n* (%) for categorical data. The cases available for each analysis are indicated in each column

**Table 3:** Demographic characteristics of the study population, separated by sex

	Subjects with AHT (*n* = 1341, 68.0%)			Control group (*n* = 631, 32.0%)		
	Female (*n* = 569, 42.4%)	Male (*n* = 772, 57.6%)	*p*	Female (*n* = 307, 48.7%)	Male (*n* = 324, 51.3%)	*p*
Clinical Parameters						
Age (years)	67 (60–72) *N* = 569	67 (60–71) *N* = 772	0.181	59 (52–67) *N* = 307	62 (54–68) *N* = 324	< 0.001
BMI (kg/m^2^)	26.7 (23.5–30.1) *N* = 537	27.3 (25.0–29.8) *N* = 746	0.010	24.1 (21.9–27.1) *N* = 292	25.6 (23.3–27.9) *N* = 311	< 0.001
Resting heart rate (bpm)	70 (62–78) *N* = 279	69 (61–76) *N* = 391	0.098	69 (63–75) *N* = 159	64 (58–71) *N* = 141	< 0.001
Systolic resting blood pressure (mmHg)	148 (19) *N* = 295	150 (18) *N* = 415	0.069	124 (11) *N* = 142	127 (8) *N* = 167	0.002
Diastolic resting blood pressure (mmHg)	85 (9) *N* = 293	86 (11) *N* = 410	0.091	76 (7) *N* = 160	78 (7) *N* = 153	0.082
Place of birth (European country)	548 (96.3) *N* = 569	748 (96.9) *N* = 772	0.559	295 (96.1) *N* = 307	303 (90.7) *N* = 311	0.731
Cardiovascular Risk Factors						
Diabetes mellitus	54 (10.1) *N* = 534	90 (12.3) *N* = 734	0.234	7 (2.5) *N* = 283	9 (3.1) *N* = 290	0.647
Active Smoking	93 (16.3) *N* = 565	124 (16.1) *N* = 771	0.854	59 (19.2) *N* = 307	76 (23.5) *N* = 324	0.194
Hyperlipoproteinemia	110 (20.4) *N* = 538	208 (28.5) *N* = 729	< 0.001	28 (9.9) *N* = 284	54 (18.4) *N* = 294	0.003
Medication						
Intake of Medication	492 (89.1) *N* = 552	601 (79.8) *N* = 753	< 0.001	224 (76.5) *N* = 293	189 (63.4) *N* = 298	< 0.001
Lipid-lowering Medication, *n*	95 (17.2) *N* = 552	145 (19.3) *N* = 753	0.346	18 (6.1) *N* = 293	25 (8.4) *N* = 298	0.293
Antihypertensive Medication, *n*	262 (47.5) *N* = 552	325 (43.2) *N* = 753	0.123	/	/	/
Antidiabetic Medication, *n*	40 (7.2) *N* = 552	64 (8.5) *N* = 753	0.409	4 (1.4) *N* = 293	7 (2.3) *N* = 298	0.376
Anticoagulant Medication, *n*	65 (11.8) *N* = 552	107 (14.2) *N* = 753	0.199	8 (2.7) *N* = 293	22 (7.4) *N* = 298	0.010
Laboratory Parameters						
Hemoglobin (g/dL)	13.8 (0.9) *N* = 551	14.9 (1.0) *N* = 751	< 0.001	13.6 (0.9) *N* = 300	14.9 (1.0) *N* = 318	< 0.001
Hematocrit (vol%)	41.5 (2.6) *N* = 551	44.9 (15.7) *N* = 751	< 0.001	41.0 (2.7) *N* = 300	44.3 (2.9) *N* = 318	< 0.001
GFR (mL/min)	85.1 (77.6–90.3) *N* = 515	89.7 (83.4–94.9) *N* = 723	< 0.001	89.4 (82.8–95.5) *N* = 277	92.6 (86.0–99.0) *N* = 304	< 0.001
Glucose (mg/dL)	93.0 (87.0–101.0) *N* = 555	96.5 (89.0–104.0) *N* = 746	< 0.001	88.0 (83.0–93.0) *N* = 299	92.0 (87.0–98.5) *N* = 317	< 0.001
HbA1c (%)	5.6 (5.4–5.9) *N* = 548	5.6 (5.4–5.9) *N* = 749	0.621	5.4 (5.2–5.6) *N* = 299	5.5 (5.3–5.7) *N* = 318	0.006
Cholesterol (mg/dL)	221.9 (42.5) *N* = 557	203.7 (39.6) *N* = 750	< 0.001	221.7 (38.2) *N* = 299	205.0 (37.1) *N* = 317	< 0.001
HDL (mg/dL)	70.0 (58.0–82.0) *N* = 548	53.0 (46.0–66.0) *N* = 750	< 0.001	73.0 (65.0–88.0) *N* = 299	55.0 (47.0–68.0) *N* = 317	< 0.001
LDL (mg/dL)	127.9 (38.1) *N* = 552	120.9 (35.7) *N* = 740	< 0.001	126.4 (35.2) *N* = 298	123.0 (33.8) *N* = 314	0.228
Triglycerides (mg/dL)	99.0 (76.0–132.0) *N* = 557	115.0 (85.0–157.5) *N* = 749	< 0.001	82.0 (63.0–111.0) *N* = 299	101.0 (72.3–141.0) *N* = 316	< 0.001
Nt-proBNP (ng/L)	118.0 (66.0–204.0) *N* = 556	65.5 (40.0–117.0) *N* = 754	< 0.001	81.0 (50.0–122.3) *N* = 298	55.5 (32.0–93.8) *N* = 316	< 0.001
Troponin (pg/mL)	2.1 (1.5–3.1) *N* = 519	3.0 (2.0–4.6) *N* = 724	< 0.001	1.5 (1.0–2.0) *N* = 277	2.3 (1.6–3.3) *N* = 306	< 0.001

Numbers are mean ± SD or median (interquartile range) for continuous and *n* (%) for categorical data. The cases available for each analysis are indicated in each column

*BMI* body mass index, *GFR* glomerular filtration rate, *HDL* high density lipoprotein, *LDL* low density lipoprotein, *hsCRP* high-sensitivity C-reactive protein, *Nt-proBNP* N-terminal pro-B-type natriuretic peptide

The average blood pressure of patients with AHT was 149 ($\pm$ 18)/85 ($\pm$10) mmHg compared to a blood pressure of 126 ($\pm$ 10)/77 ($\pm$ 7) mmHg in the control group (*p* < 0.001). 45% of patients with AHT took antihypertensive medication. Their average blood pressure (145 $\pm$ 20/ 83 $\pm$ 10) was higher than in the control group (*p* < 0.001) but lower than in AHT patients without medication (152 $\pm$ 17/87 $\pm$ 10) (*p* < 0.001).

### Ventricular morphology, function and tissue characteristics in females vs. males

As seen in Table 4, females overall (with and without AHT) showed a higher LVEF (regression coefficient (B = +2.38%, *p* < 0.001) and RVEF (B = +3.89%, *p* < 0.001) than males. LVEDMi was lower in females (B = −19.75%, *p* < 0.001). EDVi were lower in females (B = −5.48 mL/m^2^ for the LV and −10.45 mL/m^2^ for the RV), as were ESVi (B = −3.08 mL/m^2^ for the LV and −6.84 mL/m^2^ for the RV), resulting in lower SVi (B = −2.41 mL/m^2^ for the LV and −3.70 mL/m^2^ for the RV) (all *p* < 0.001). The LVCI was not different to males (*p* = 0.233), while the RVCI was lower compared to males (B = −0.16 L/min/m^2^, *p* = 0.002). Septal midventricular T2 (B = +1.08 ms, *p* < 0.001) and T1 (B = +22.08 ms, *p* < 0.001) relaxation times were prolonged in females compared to males. Females were less likely to show LGE (B = −0.73 [95% CI: −1.15 to −0.30], *p* < 0.001, *n* = 791) than males.

**Table 4:** Results of the regression analysis, showing the effects of sex and hypertension on structural and functional CMR-parameters

Parameter	Regression coefficient (B)	95% confidence interval (95% CI)	*p*
LVEF (%) (*n* = 1691)			
Hypertension	+1.15	0.29 to 2.00	0.009
Female Sex	+2.38	1.69 to 3.08	< 0.001
(ln)LVEDMi (%) (*n* = 1690)			
Hypertension	+6.61	4.29 to 8.98	< 0.001
Female Sex	−19.75	−21.26 to −18.46	< 0.001
LVEDVi (mL/m^2^) (*n* = 1689)			
Hypertension	−0.64	−2.30 to 1.03	0.453
Female Sex	−5.48	−6.84 to −4.13	< 0.001
LVESVi (mL/m^2^) (*n* = 1682)			
Hypertension	−0.92	−1.71 to −0.12	0.024
Female Sex	−3.08	−3.72 to −2.43	< 0.001
LVSVi (mL/m^2^) (*n* = 1688)			
Hypertension	+0.28	−0.88 to 1.44	0.637
Female Sex	−2.41	−3.35 to −1.46	< 0.001
LVCI (L/min/m^2^) (*n* = 843)			
Hypertension	+0.19	0.08 to 0.30	< 0.001
Female Sex	−0.05	−0.14 to 0.04	0.233
RVEF (%) (*n* = 1674)			
Hypertension	+1.25	0.08 to 2.43	0.037
Female Sex	+3.89	2.93 to 4.84	< 0.001
RVEDVi (mL/m^2^) (*n* = 1678)			
Hypertension	−1.21	−3.07 to 0.66	0.204
Female Sex	−10.45	−11.98 to −8.94	< 0.001
RVESVi (mL/m^2^) (*n* = 1678)			
Hypertension	−1.34	−2.43 to −0.26	0.015
Female Sex	−6.84	−7.72 to −5.96	< 0.001
RVSVi (mL/m^2^) (*n* = 1670)			
Hypertension	+0.08	−1.24 to 1.40	0.902
Female Sex	−3.70	−4.78 to −2.63	< 0.001
RVCI (L/min/m^2^) (*n* = 825)			
Hypertension	+0.16	0.04 to 0.29	0.012
Sex	−0.16	−0.27 to −0.06	0.002
Septal midventricular T2 relaxation times (ms) (*n* = 1644)			
Hypertension	−0.89	−1.17 to −0.61	< 0.001
Sex	+1.08	0.85 to 1.31	< 0.001
Septal midventricular T1 relaxation times (ms) (*n* = 1620)			
Hypertension	−2.83	−7.51 to 1.86	0.237
Sex	+22.08	18.28 to 25.89	< 0.001

*LV* left ventricle, *RV* right ventricle, *EF* ejection fraction, *EDMi* end-diastolic mass index, *EDVi* end-diastolic volume index, *ESVi* end-systolic volume index, *SVi* stroke volume index, *CI* cardiac index

### Ventricular morphology, function and tissue characteristics in subjects with AHT vs. without AHT

Table 4 also displays the comparison between individuals with AHT and controls. LVEF (B = +1.15%, *p* = 0.009) and RVEF (B = +1.25%, *p* = 0.037) were elevated in subjects with AHT compared to controls. LVEDMi was also higher (B = +6.61%, *p* < 0.001). Similarly, the LVCI (B = +0.19 L/min/m^2^, *p* < 0.001) and RVCI (B = +0.16 L/min/m^2^, *p* = 0.012) were elevated in patients with AHT. T2 relaxation times were shorter in patients with AHT (B = −0.89, *p* < 0.001), while T1 relaxation times were not different (*p* = 0.237) to controls. The prevalence of LGE between participants with AHT and controls was similar (B = +0.16 [95% CI: −0.30 to 0.63], *p* = 0.392, *n* = 791). No statistically relevant changes were observed in the above-described differences between subjects with AHT and controls after excluding subjects with LGE (Supplementary Table 1). Supplementary Table 2 displays the association between other independent variables with sex and AHT.

### Sex-specific ventricular morphology, function and tissue characteristics

Table 5 shows the sex-specific marginal mean (standard error) CMR parameters in healthy controls and subjects with AHT. LVEF and RVEF were highest in females with AHT, while EDVi and ESVi were lowest in females with AHT. SVi and CI were highest in males with AHT. Males with AHT showed the highest LVEDMi, followed by male controls. Female controls showed the lowest LVEDMi.

**Table 5:** Marginal mean (standard error) CMR parameters, with respect to the effect of sex and AHT

Parameter	Marginal mean	Standarderror	*p*
LVEF (%) (*n* = 1691)			
Male Control	67.5	0.9	
Male + AHT	68.5	0.8	0.066
Female Control	69.7	0.9	< 0.001
Female + AHT	71.0	0.8	< 0.001
LVEDMi (g/m^2^) (*n* = 1690)			
Male Control	64.7	1.4	
Male + AHT	68.7	1.2	< 0.001
Female Control	50.8	1.4	< 0.001
Female + AHT	55.1	1.3	< 0.001
LVEDVi (mL/m^2^) (*n* = 1689)			
Male Control	59.8	1.7	
Male + AHT	59.2	1.5	0.576
Female control	54.4	1.7	< 0.001
Female + AHT	53.7	1.5	< 0.001
LVESVi (mL/m^2^) (*n* = 1682)			
Male Control	19.6	0.8	
Male + AHT	18.7	0.8	0.095
Female Control	16.6	0.8	< 0.001
Female + AHT	15.6	0.8	< 0.001
LVSVi (mL/m^2^) (*n* = 1688)			
Male Control	40.1	1.2	
Male + AHT	40.4	1.0	0.744
Female Control	37.6	1.2	0.004
Female + AHT	38.0	1.2	0.007
LVCI (L/min/m^2^) (*n* = 843)			
Male Control	2.6	0.1	
Male + AHT	2.7	0.1	0.045
Female Control	2.5	0.1	0.126
Female + AHT	2.7	0.1	0.114
RVEF (%) (*n* = 1674)			
Male Control	54.5	1.2	
Male + AHT	55.2	1.0	0.314
Female Control	57.6	1.2	< 0.001
Female + AHT	59.4	1.0	< 0.001
RVEDVi (mL/m^2^) (*n* = 1678)			
Male Control	64.0	1.9	
Male + AHT	62.9	1.6	0.347
Female Control	53.7	1.9	< 0.001
Female + AHT	52.4	1.7	< 0.001
RVESVi (mL/m^2^) (*n* = 1678)			
Male Control	29.3	1.1	
Male + AHT	28.1	1.0	0.090
Female Control	22.7	1.1	< 0.001
Female + AHT	21.2	1.0	< 0.001
RVSVi (mL/m^2^) (*n* = 1670)			
Male Control	34.9	1.3	
Ma le + AHT	34.9	1.2	0.940
Female Control	31.1	1.3	< 0.001
Female + AHT	31.2	1.2	< 0.001
RVCI (L/min/m^2^) (*n* = 825)			
Male Control	2.3	0.1	
Male + AHT	2.4	0.1	0.258
Female Control	2.0	0.1	0.004
Female + AHT	2.2	0.1	0.798

Male controls are the reference category in the model

*LV* left ventricle, *RV* right ventricle, *EF* ejection fraction, *EDMi* end-diastolic mass index, *EDVi* end-diastolic volume index, *ESVi* end-systolic volume index, *SVi* stroke volume index, *CI* cardiac index

**Fig. 2:**
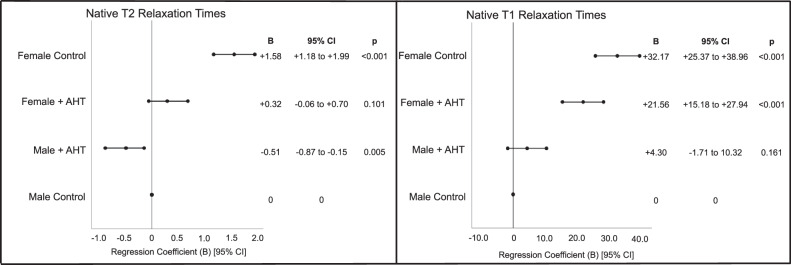
Forrest plot showing the sex-specific differences in native septal midventricular T2 and T1 relaxation times between subjects with AHT and controls, after excluding LGE-positive segments. Compared to male controls (as reference category in this model), female controls showed the longest septal midventricular native T1 and T2 relaxation times, followed by females with AHT. Males with AHT showed similar T1 relaxation times to male controls but slightly lower T2 relaxation times

**Fig. 3:**
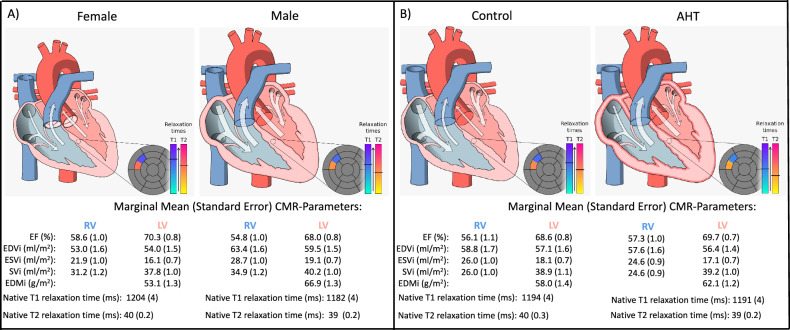
Schematic illustration on the effects of (**A**) sex and (**B**) AHT on ventricular volumes, function, and tissue characteristics in the HCH-study. Ventricular end-diastolic (represented by the arrows from the atria to the ventricles) and end-systolic volumes, as well as stroke volumes (represented by the arrows from the ventricles to the vessels) and mass (represented by myocardial thickness) were lower in females compared to males. Subjects with AHT showed a higher ventricular mass than controls. Midventricular native septal T1 and T2 relaxation times were longer in females than in males. Patients with AHT showed shorter T2 and similar T1 relaxation times to controls

To analyze the interaction between sex and AHT on septal midventricular T1 and T2 relaxation times, subjects with midventricular septal LGE (*n* = 31) were excluded. The interactive effect of AHT and sex on native T1 (*p* < 0.001) and T2 relaxation times (*p* = 0.003) was significant (Fig. 2). In males, T2 relaxation times were shorter in subjects with AHT (B = −0.68 [−1.04 to −0.31], *p* < 0.001, *n* = 647), while T1 relaxation times were not significantly different to controls (B = +1.47 [−5.09 to 8.02], *p* = 0.661, *n* = 821). Similarly, females with AHT also showed shorter T2 relaxation times (−1.11 [−1.59 to −0.63], *p* < 0.001, *n* = 653) than female controls, while T1 relaxation was not significantly different (−4.27 [−11.69 to 3.15], *p* = 0.259, *n* = 840). Figure 3 schematically displays the effect of sex and AHT on ventricular morphology, function, and tissue characteristics in the HCH-study.

## Discussion

The main findings of this CMR-derived observational analysis of the HCH-study population were as follows:

1. Females overall showed a higher EF, but lower volumes, LV mass, and CI compared to males. Native septal midventricular T1 and T2 relaxation times were longer in females than in males.
2. Subjects with AHT showed a higher EF, CI, and LV mass compared to healthy controls, independent of sex.
3. Differences in midventricular native T1 and T2 relaxation times between patients with AHT and controls appeared as sex-specific.

Female participants of the HCH study overall showed lower volumes and left ventricular end-diastolic mass than males after adjusting for age, BMI, place of birth, medication, and cardiovascular risk factors. Despite lower volumes, LV- and RVEF were higher in females. Similar observations were made in other population-based cohort studies, such as the “Dallas Heart Study” [13] and the “UK Biobank population cohort” [14].

Subjects with AHT showed a higher LVEDMi, as well as marginally higher EF and CI compared to controls. The average blood pressure of subjects with AHT in this cohort was 148.9 $(\! \pm 18.4)$ (*n* = 716)/85.1 $(\! \pm 10.2)$ (*n* = 698) mmHg, corresponding to Grade I hypertension [10]. Hence, it can be assumed that most of the patients investigated here were experiencing an early stage of cardiac remodeling with predominant cardiomyocyte hypertrophy [15, 16]. Cellular hypertrophy, as the first step in adaptive remodeling as a response to AHT, primarily leads to a reduction in extracellular volume [17]. This could explain why T1 relaxation times were not significantly elevated and why T2 relaxation times were even minimally shorter in subjects with AHT compared to healthy controls in this study. Correspondingly, there was no significant increase in the likelihood of LGE in HCHS participants with AHT. Similar to our results, only a minority of otherwise asymptomatic patients with treated AHT demonstrated LGE in previous literature [16, 18]. Therefore, it has recently been suggested to employ LGE and T1 mapping to discriminate between hypertensive heart disease (normal or slightly elevated T1 relaxation, overlap with controls) and hypertrophic cardiomyopathy (elevated T1 relaxation times and increased prevalence of LGE) [19–21]. Cardiomyocyte hypertrophy leads to cellular apoptosis and diffuse myocardial fibrosis in the long-term [22], resulting in impaired cardiac contractility. Despite the lack of myocardial fibrosis in this study, the described effects of AHT on structural CMR parameters could be prognostically relevant on a per-patient level. Studies reported an increase in the hazard of heart failure by 3% for each additional g/m^2^ increase in left ventricular mass index [23]. Thus, the detection of left ventricular hypertrophy at a subclinical stage in the setting of AHT would allow for optimization and monitoring of antihypertensive management before irreversible cardiac damage occurs.

When combining the effect of sex and AHT, females with AHT showed the highest marginal mean LV and RVEF, while simultaneously having the lowest volumes. Previous echocardiographic studies also found a higher EF in hypertensive women compared to men [7]. The generally elevated EF observed in females, which further increased in the setting of AHT, could explain why females are often diagnosed with “heart failure with preserved ejection fraction” (HFpEF) than males in the setting of risk factors, such as hypertension [7]. These findings stress the necessity of interpreting the EF in combination with volumes and mass [24]. Sex-specific EF cut-off values should help to detect ventricular dysfunction sooner in females (especially with AHT), allowing females to qualify for proven treatments when needed [25].

Contradictory to our results, where the association between AHT and increase in LVEDMi was similar in males and females, few previous echocardiographic studies reported a higher prevalence of left ventricular hypertrophy in females versus males with AHT [7, 26] while others reported lower odds for LV hypertrophy in females [27]. Compared to most previous literature, this analysis investigates asymptomatic subjects with predominantly mild or treated AHT, representing the majority of AHT patients in Germany [28]. What further distinguishes this analysis from echocardiographic studies is that CMR enables more accurate and observer-independent assessment of cardiac structure and function, also allowing analysis of RV remodeling, which has so far only been investigated in small patient groups with similar results as reported here [29].

Furthermore, this is the first study that investigates sex-specific tissue characteristics in the setting of AHT, to our knowledge. Females overall showed longer T2 and T1 relaxation times compared to males. Some previous studies reported prolonged T1 relaxation times in healthy females compared to males at 1.5 and 3 T, independent of age [30–32]. Sex-specific T2 values, on the other hand, have only been investigated in small studies including healthy volunteers [30, 33], with contradictory results. To some extent, sex-specific differences in myocardial T1 and T2 relaxation times might be caused by differences in the relaxation time of the blood pool, which is in turn influenced by a range of factors, including hematocrit and oxygen saturation [34, 35]. Hematocrit was lower in females compared with males in this study, which could explain the prolonged myocardial T1 relaxation times in females [36]. Beyond that, females were less likely to show LGE in this cohort, suggesting that the prolonged T1 relaxation in females does not imply increased myocardial fibrosis. On the contrary, many anti-inflammatory hormonal and cellular pathways have been described in the female heart [37].

AHT was associated with shortened T2 relaxation times in both males and females, but the effect was more prominent in females. As described above, AHT might be associated with shortened T2 relaxation times due to a reduction in extracellular volume as a result of cellular hypertrophy [17]. Some previous echocardiographic studies reported a more prevalent left ventricular hypertrophy in untreated females with AHT compared to males, which was less modifiable by antihypertensive treatment [7] and supports this theory [38]. On the other hand, the effect of LVEDMi did not appear sex-specific in this analysis, possibly because the patients in this study were at an early stage of remodeling. It remains to be elucidated, how exactly sex affects multiparametric mapping in various diseases.

Although aging is known to impact cardiac structure and function as well as tissue characteristics by T1 and T2 mapping and LGE, the effect of age was not separately investigated in this study, because the subgroups were not of a representative size and the participants in the HCH study only represent the age groups between 45 and 74 years. Only 44.4% of study participants received a contrast agent, so the LGE analysis could only be performed in a fraction of the study population, also leading to a possible selection bias. Furthermore, the study was only performed at 3 T, which needs to be considered when interpreting T1 and T2 relaxation times in particular. As an inherent limitation of population-based studies, data was missing for each parameter. To achieve the most reliable results, variable-wise analyses were performed, and the number of available complete cases was noted for each variable.

To conclude, it is well known that sex affects blood pressure regulation and pathophysiology of AHT, as well as complications and treatment response [2]. However, only little is known about possible sex-specific differences in structural and functional heart changes as a response to AHT. This study provides added value to current knowledge, as it portrays sex-specific changes in structural and functional CMR parameters as a response to AHT. Study participants with AHT showed increased ventricular mass, marginally increased EF and cardiac output, and shorter native T2 relaxation times than controls. The EF was highest in females with AHT, while stroke volumes were highest in males with AHT. Furthermore, T1 and T2 relaxation times appeared sex-specific in patients with AHT. Females overall showed lower septal midventricular T1 and T2 relaxation times. AHT was associated with a shortening in T2 relaxation, which appeared more prominent in females than males. These findings are necessary to consider, as CMR is gaining importance in the assessment of hypertensive heart disease, for example in treatment studies or to differentiate between hypertensive heart disease and other hypertrophic conditions like hypertrophic cardiomyopathy. When interpreting CMR exams regarding possible hypertensive heart disease, it is important to note that indicators of myocardial fibrosis, such as T1 prolongation and LGE, are often not present during early remodeling (as in this study) and that T2 relaxation times might even be shortened in subjects with AHT, especially in females. Furthermore, females overall show a comparatively high EF, which could explain why especially females are often diagnosed with HFpEF in the setting of AHT. Also, females show prolonged T1 and T2 relaxation times, which needs to be considered when establishing and interpreting cut-off values for pathological T1 and T2 relaxation times.

## Supplementary information

Electronic Supplementary Material

## Abbreviations

AHT: Arterial hypertension
BMI: Body mass index
CI: Cardiac index
CMR: Cardiac magnetic resonance imaging
EDMi: End-diastolic mass index
EDVi: End-diastolic volume index
EF: Ejection fraction
ESVi: End-systolic volume index
GFR: Glomerular filtration rate
HCHS: Hamburg City Health Study
HDL: High density lipoprotein
HFpEF: Heart failure with preserved ejection fraction
LDL: Low density lipoprotein
LV: Left ventricle
Nt-proBNP: N-terminal pro-B-type natriuretic peptide
RV: Right ventricle
SVi: Stroke volume index
