# Supplementary material for: Sex-specific ventricular morphology, function, and tissue characteristics in arterial hypertension: a magnetic resonance study of the Hamburg city health cohort
Source: Eur Radiol. 2024 May 31;34(11):7309–20. doi: 10.1007/s00330-024-10797-2 (PMC11519140; doi:10.1007/s00330-024-10797-2)
Supplement: Supplementary file 1 — Electronic Supplementary Material [file 330_2024_10797_MOESM1_ESM.pdf]

Sex-specific ventricular morphology, function, and tissue characteristics in arterial hypertension: A magnetic resonance study of the Hamburg city health cohort

Electronic Supplementary Material (ESM)

Eur Radiol (2024) Erley J, Jahnke CM, Schüttler S et al.

Supplementary Table 1: Results of the regression analysis, showing the effects of sex and AHT on structural and functional CMR-parameters, after excluding subjects with LGE.

Parameter	Regression coefficient (B)	95% confidence interval (95% CI)	*P*
**LVEF (%) (n=1419)**			
Hypertension	+1.32	0.39 to 2.24	0.005
Female Sex	+2.16	1.41 to 2.91	<0.001
**(ln)LVEDMi (%) (n=1418)**			
Hypertension	+6.18	3.67 to 8.65	<0.001
Female Sex	-19.75	-20.94 to -17.88	<0.001
**LVEDVi (ml/m^2) (n=1418)**			
Hypertension	-0.43	-2.21 to 1.35	0.638
Female Sex	-5.30	-6.74 to -3.86	<0.001
**LVESVi (ml/m^2) (n=1413)**			
Hypertension	-0.94	-1.80 to -0.09	0.031
Female Sex	-2.84	-3.53 to -2.15	<0.001
**LVSVi (ml/m^2) (n=1417)**			
Hypertension	+0.53	-7.1 to 1.77	0.404
Female Sex	-2.44	-3.44 to -1.44	<0.001
**LVCI (l/min/m^2) (n=742)**			
Hypertension	+0.21	0.09 to 0.33	<0.001
Female Sex	-0.07	-0.16 to 0.03	0.178

**Parameter**	**Regression coefficient (B)**	**95% confidence interval (95% CI)**	***P***
**RVEF (%) (n=1403)**			
Hypertension	+1.29	0.01 to 2.58	0.049
Female Sex	+3.95	2.91 to 4.99	<0.001
**RVEDVi (ml/m^2) (n=1408)**			
Hypertension	-1.23	-3.23 to 0.78	0.231
Female Sex	-10.77	-12.39 to -9.14	<0.001
**RVESVi (ml/m^2) (n=1408)**			
Hypertension	-1.35	-2.53 to -0.18	0.024
Female Sex	-7.05	-8.00 to -6.09	<0.001
**RVSVi (ml/m^2) (n=1401)**			
Hypertension	+0.11	-1.32 to 1.53	0.885
Female Sex	-3.78	-4.94 to -2.63	<0.001
**RVCI (l/min/m^2) (n=726)**			
Hypertension	+0.19	0.05 to 0.32	0.006
Sex	-0.18	-0.29 to -0.08	<0.001
**Septal midventricular T2 relaxation times (ms) (n=1386)**			
Hypertension	-0.91	-1.22 to -0.61	<0.001
Sex	+1.02	0.77 to 1.27	<0.001
**Septal midventricular T1 relaxation times (ms) (n=1355)**			
Hypertension	-1.74	-6.90 to 3.43	0.510
Sex	+22.78	18.61 to 26.96	<0.001

Abbreviations: AHT = arterial hypertension, LV = left ventricle, RV = right ventricle, EF = ejection fraction, EDMi = end-diastolic mass index, EDVi = end-diastolic volume index, ESVi = end-systolic volume index, SVi = stroke volume index, CI = cardiac index.

Supplementary Table 2: Results of the regression analysis, showing the effects of other independent variables on structural and functional CMR-parameters. Only statistically significant results are displayed.

**Parameter**	**Regression coefficient (B)**	**95% confidence interval (95% CI)**	***P***
**LVEF (%) (n=1691)**			
Antihypertensive medication	+1.73	0.87 to 2.58	<0.001
**(ln)LVEDMi (%) (n=1690)**			
Diabetes mellitus	-0.07	-0.13 to -0.01	0.016
Smoking	+0.04	0.01 to 0.06	0.002
Age	-0.001	-0.003 to 0.00	0.012
BMI	+0.003	0.0 to +0.005	0.017
**LVEDVi (ml/m^2) (n=1689)**			
Diabetes mellitus	-4.66	-9.16 to -0.02	0.042
Smoking	-3.80	-5.59 to -2.02	<0.001
Hyperlipoproteinemia	-3.72	-6.60 to -0.84	0.011
Age	-0.41	-0.49 to -0.32	<0.001
BMI	-0.43	-0.59 to -0.27	<0.001
**LVESVi (ml/m^2) (n=1682)**			
Smoking	-0.97	-1.82 to -0.11	0.026
Antihypertensive medication	-1.12	-1.91 to -0.32	0.006
Age	-0.13	-0.17 to -0.09	<0.001
BMI	-0.14	-0.22 to -0.07	<0.001
**LVSVi (ml/m^2) (n=1688)**			
Smoking	-2.83	-4.07 to -1.58	<0.001
Hyperlipoproteinemia	-2.29	-4.30 to -0.28	0.025
Antihypertensive medication	+1.18	0.02 to 2.35	0.046

Age	-0.27	-0.33 to -0.21	<0.001
BMI	-0.29	-0.39 to -0.18	<0.001
**LVCI (l/min/m^2) (n=843)**			
Age	-0.014	-0.020 to -0.008	<0.001
BMI	-0.013	-0.024 to -0.002	0.018
**RVEF (%) (n=1674)**			
Smoking	-1.53	-2.79 to -0.03	0.017
**RVEDVi (ml/m^2) (n=1678)**			
Non-European Origin	-4.26	-8.36 to -0.16	0.042
Diabetes mellitus	-6.32	-11.35 to -1.29	0.014
Smoking	-3.05	-5.06 to -1.05	0.003
Hyperlipoproteinemia	-4.80	-8.06 to -1.54	0.004
Lipid-lowering medication	+4.03	0.23 to 7.82	0.037
Age	-0.50	-0.59 to -0.40	<0.001
BMI	-0.47	-0.65 to -0.29	<0.001
**RVESVi (ml/m^2) (n=1678)**			
Age	-0.24	-0.29 to -0.18	<0.001
BMI	-0.21	-0.32 to -0.11	<0.001
**RVSVi (ml/m^2) (n=1670)**			
Non-European Origin	-3.02	-5.94 to -0.10	0.042
Smoking	-2.49	-3.91 to -1.07	<0.001
Hyperlipoproteinemia	-3.16	-5.48 to -0.85	0.007
Lipid-lowering medication	2.92	0.23 to 5.60	0.034
Age	-0.26	-0.32 to -0.19	<0.001
BMI	-0.27	-0.39 to -0.14	<0.001
**RVCI (l/min/m^2) (n=825)**			

Diabetes mellitus	-0.32	-0.64 to -0.10	0.043
Antihypertensive medication	-0.17	-0.29 to -0.05	0.007
Antidiabetic medication	+0.37	0.002 to 0.73	0.048
Anticoagulant medication	-0.20	-0.37 to -0.03	0.022
Age	-0.01	-0.02 to -0.004	<0.001
BMI	-0.02	-0.03 to -0-003	0.018
**Septal midventricular T2 relaxation times (ms) (n=1644)**			
Hyperlipoproteinemia	-0.50	-0.99 to -0.01	0.046
BMI	-0.04	-0.07 to -0.01	0.005
**Septal midventricular T1 relaxation times (ms) (n=1620)**			
Smoking	+9.70	4.62 to 14.78	<0.001
Antihypertensive medication	-4.72	-9.41 to -0.03	0.049

Abbreviations: LV = left ventricle, RV = right ventricle, EF = ejection fraction, EDMi = end-diastolic mass index, EDVi = end-diastolic volume index, ESVi = end-systolic volume index, SVi = stroke volume index, CI = cardiac index.
